# Parkinson’s disease dementia and hearing impairment, are they related? A UK biobank pilot analysis

**DOI:** 10.1007/s00221-026-07282-1

**Published:** 2026-04-09

**Authors:** Megan Rose Readman, Clarissa Giebel, Jacqui Cannon, Ian Fairman, Christopher J. Plack

**Affiliations:** 1 Department of Primary Care and Mental Health, The University of Liverpool, Waterhouse Building Block B, 2 Floor, Liverpool, L69 3GL UK; 2 National Institute for Health and Care Research Applied Research Collaboration North West Coast, Liverpool, UK; 3 Department of Psychology, Lancaster University, Lancaster, UK; 4 The Lewy Body Society, Wigan, UK; 5 Manchester Centre for Audiology and Deafness, The University of Manchester, Manchester, UK

**Keywords:** Hearing loss, Dementia, Risk, Parkinson’s disease dementia

## Abstract

Hearing impairment is implicated as a risk factor for the incidence of all-cause dementia. However, few studies have explored the relationship between hearing loss and specific rarer dementia subtypes. Therefore, this pilot study sought to explore whether hearing impairment, estimated using a speech-in-noise test (the digit triplet test, DTT), is a risk factor for Parkinson’s disease dementia (PDD) incidence. We conducted a pre-registered prospective cohort study using UK Biobank data from 158,686 individuals (72,015 males; 86,671 females), who underwent the DTT, and were free from PDD at the point of assessment. A Cox Proportional Hazard model, controlling for age, sex and educational attainment was conducted. Over a median follow-up of 8.93 years (SD = 2.29), 101 cases of incident PDD were observed. While the hazard ratio were in the direction of a positive relationship between baseline DTT and PDD incidence, imprecision in estimates leaves it uncertain as to whether risk was influenced by speech-reception-threshold (HR = 2.39, 95% CI: 0.75,7.63; *p* = .141), and findings should be treated as exploratory. An exploratory analysis, in which speech-reception-thresholds were categorised according to UK Biobank norms, showed that ‘insufficient’ (*p* = .024), but not ‘poor’ (*p* = .236) hearing increased PDD risk. The observed HR directionally aligns with the previously observed positive relationship between hearing loss and all-cause dementia incidence. However, further studies employing alternative datasets with more PDD cases are required to substantiate these findings. If further supported, these findings may have clinical and public health implications.

**Supplementary Information:**

The online version contains supplementary material available at 10.1007/s00221-026-07282-1.

## Background

Whilst not traditionally considered a cardinal feature of Parkinson’s disease (Parkinson’s), cognitive impairment and dementia are increasingly being recognised as common non-motor features of Parkinson’s (Degirmenci et al. 2023). Indeed, population based epidemiological studies estimate point prevalence of dementia in Parkinson’s, also referred to as Parkinson’s disease dementia (PDD), to be between 20 and 30% (Sousa et al. 2022; Aarsland et al. 2005). Moreover, PDD is thought to account for ~ 4% of all dementia cases (Aarsland et al. 2005).

Symptomatically, PDD is largely heterogeneous. However, typical presentation incorporates the core extrapyramidal symptoms that hallmark Parkinson’s (e.g. bradykinesia, tremor, rigidity and postural instability (Degirmenci et al. 2023)) accompanied by cognitive, behavioural and psychiatric symptoms (Goetz et al. 2008). The cognitive manifestation typically includes deficits in the domains of attention, short term memory, cued memory recall, executive function, visuospatial function and language, and fluctuations in cognitive function (Goetz et al. 2008), and behavioural and psychiatric symptoms include hallucinations (particularly in the visual domain), delusions, mood disturbances, and apathy (Goetz et al. 2008).

Growing evidence has independently implicated hearing loss as a substantial risk factor for the incidence of Parkinson’s (Schrag et al. 2023, Lai et al. 2014, Simonet et al. 2022, Readman et al. 2025a, Readman et al. 2023) and all-cause dementia (Readman et al. 2025b). Specifically, clinically diagnosed hearing loss increases the risk of Parkinson’s incidence 1.5–1.6 fold over 2–5 years follow up (Lai et al. 2014; Simonet et al. 2022), and every 10 dB worsening in speech-reception threshold (SRT) is associated with a 57% increase in Parkinson’s incidence risk [Hazard ratio (HR): 1.57 (95%CI: 1.018, 2.435; *P* = 0.041)] (Readman et al. 2025a). Considering dementia, the presence of hearing loss (Yes vs. No) is associated with increased risk of incident dementia [ HR = 1.32 [95% CI: 1.23–1.41]], and every 10 dB worsening of SRT is associated with an approximate 20% increase in dementia risk [HR = 1.26 [95% CI: 0.89; 1.78] (Readman et al. 2025b). Moreover, hearing loss was identified as one of 14 clinical conditions/ lifestyle factors that are risk factors for dementia in the 2024 Lancet report with a Population Attributable Risk Factor of 7% (Livingston et al. 2024).

The independent studies contributing to prior meta-analyses and the Lancet report provide important insights regarding dementia risk reduction. However, most studies to date have employed an all-cause dementia outcome variable. In accordance with the World Health Organisation definition (Mehta and Schneider 2023), dementia is not a single condition but rather is an umbrella term used to refer to a group of heterogeneous neurological conditions that feature symptoms affecting memory, thinking, and social abilities. It is presently estimated that there are over 200 different subtypes of dementia, which all differ in terms of prevalence, symptomology, and pathophysiology (Mehta and Schneider 2023). Therefore, in employing an all-cause dementia category as the outcome variable, it remains unclear whether hearing loss is a universal risk factor for all types of dementia or whether hearing loss poses a differential risk dependent upon dementia subtype. Specifically, as Alzheimer’s disease dementia (AD) accounts for between 60 and 80% of all dementia cases (Alzheimer’s Association 2024), it is possible that the statistical relations observed in prior studies are predominantly reflective of AD. Therefore, based upon the prior studies it remains unclear whether peripheral auditory deficit is a risk factor for incident PDD.

Several hypotheses have been proposed to account for the relationship between hearing loss and dementia. One of these hypotheses, the common cause hypothesis, asserts that a common pathology gives rise to both hearing loss (by affecting the cochlea and ascending auditory pathway) and dementia (by affecting by the cortex) (Griffiths et al. 2020). Two potential candidates for the common neuropathological cause are mitochondrial oxidative stress (Paciello et al. 2023) and alterations in the production, and subsequent aggregation, of α-Synuclein (Hamilton 2000, Park et al. 2011). Alternative hypotheses include the sensory deprivation and information degradation hypotheses (Griffiths et al. 2020). The sensory deprivation hypothesis postulates that prolonged auditory deprivation, due to hearing impairment, may give rise to cortical reorganization and altered brain structures in the auditory cortex and hippocampus that hinders cognitive processes in favour of auditory perception (Powell et al. 2022). Alternatively, the information degradation hypothesis postulates that when hearing impairment occurs, greater effort (‘listening effort’) is required for the individual to be able to accurately process and comprehend the auditory signal (Griffiths et al. 2020). This results in a greater proportion of the fundamentally limited cognitive resources (Kahneman and Tversky 1973) being diverted towards listening and away from other cognitive tasks. Resultantly, the individual has less resources available for cognitive functions which over time may lead to cognitive decline (Humes et al. 2013).

The occurrence of cognitive impairment in Parkinson’s appears to be related to both the degree of oxidative stress and α-Synuclein Lewy body pathology experienced (Degirmenci et al. 2023). Thus, in accordance with the common cause hypothesis we may anticipate that hearing impairment will increase risk of incident PDD. Similarly, on a structural level, neuroimaging studies have shown that PDD is associated with cortical atrophy in the medial temporal lobe and inferior temporal regions (Oppedal et al. 2019; Kantarci et al. 2022), two core brain regions consistently implicated in human cognitive functioning including executive functioning (Alvarez and Emory 2006), attention (Ramezanpour and Fallah 2022), and memory (Berron et al. 2020). Thus, in accordance with the sensory deprivation hypothesis we may anticipate that hearing loss could give rise to cortical reorganisation that hinders cognitive function and hence will be a substantial risk factor for PDD. Finally, in line with the information degradation hypothesis, we may anticipate that the reallocation of finite cognitive resources to facilitate listening under circumstances of hearing loss may increase the likelihood that people will go on to experience the cognitive deficits implicated in PDD (Griffiths et al. 2020).

Limited evidence suggests that auditory dysfunction may be prevalent in PDD (see Johnson et al. (2021) for review). For example, Kofler et al. (2001) observed that people with PDD had fewer, smaller, and delayed muscular startle responses in response to acoustic tone bursts. Moreover, Brønnick et al. (2010) also observed that people with PDD displayed impaired automatic auditory change detection. These studies provide some understanding of auditory dysfunction in the context of PDD. However, it has been suggested that muscular startle responses in response to acoustic tone bursts may be influenced by inattentiveness (Kofler et al. 2001), and auditory change detection relies to some extent upon memory representations (Pekkonen et al. 1994). As such it is unclear whether the observed findings primarily reflect auditory or cognitive deficits in people with PDD. Thus, it remains unclear whether peripheral hearing loss is a risk factor for the occurrence of incident PDD. The aim of the present study was therefore to investigate whether hearing impairment is a risk factor for subsequent PDD diagnosis using data from the UK Biobank (Sudlow et al. 2015).

## Methods

This study was pre-registered on the Open Science Framework (OSF; https://osf.io/f8yh7/) (Readman et al. 2024). The data analysed in this study are available through protected. Access to the analysed data was gained following successful application to the UK Biobank (Application Number: 98097). The OSF pre-registration contains documentation of the variables analysed (including transformations), planned statistical analyses, and the data analysis code book.

### Study population

This study analysed data from the UK Biobank. The UK Biobank is an ongoing longitudinal biomedical database containing 503,325 participants aged between 40 and 69 years who were recruited between 2006 and 2010 (Sudlow et al. 2015). All participants were assessed at one of 22 locations across the UK and underwent comprehensive assessment including completion of self-report touchscreen assessment, physical examination, and provision of biological samples. Since 2009 an adaptive test of speech in noise perception, the digit-triplet test (DTT) (Smits et al. 2004), has been included in the touchscreen assessment, with 166,886 participants completing the hearing test at baseline. Further details regarding the UK Biobank protocol can be on the UK Biobank website (https://www.ukbiobank.ac.uk/media/gnkeyh2q/study-rationale.pdf).

The UK Biobank received ethical approval from the UK National Health Service (NHS) North West Multi-centre Research Ethics Committee (Ref 11/NW/038). As all data are anonymised, this secondary data analysis did not require additional ethical approval.

The present study utilised data from the baseline assessment (data collection 13/03/2006- 01/10/2010). Participants were excluded if they:

1. Had a known diagnosis of PDD at the time of completing the assessment,
2. Later went on to develop AD or PD (in the absence of Parkinson’s Dementia),
3. Were missing data for the hearing test,
4. Were missing covariate data
5. Responded incorrectly on almost every trial of the hearing test, defined as the total number of correct trials being <  = 1 or the calculated SRT being within 1 dB of the ceiling of the procedure (+ 8 dB)

**Fig. 1:**
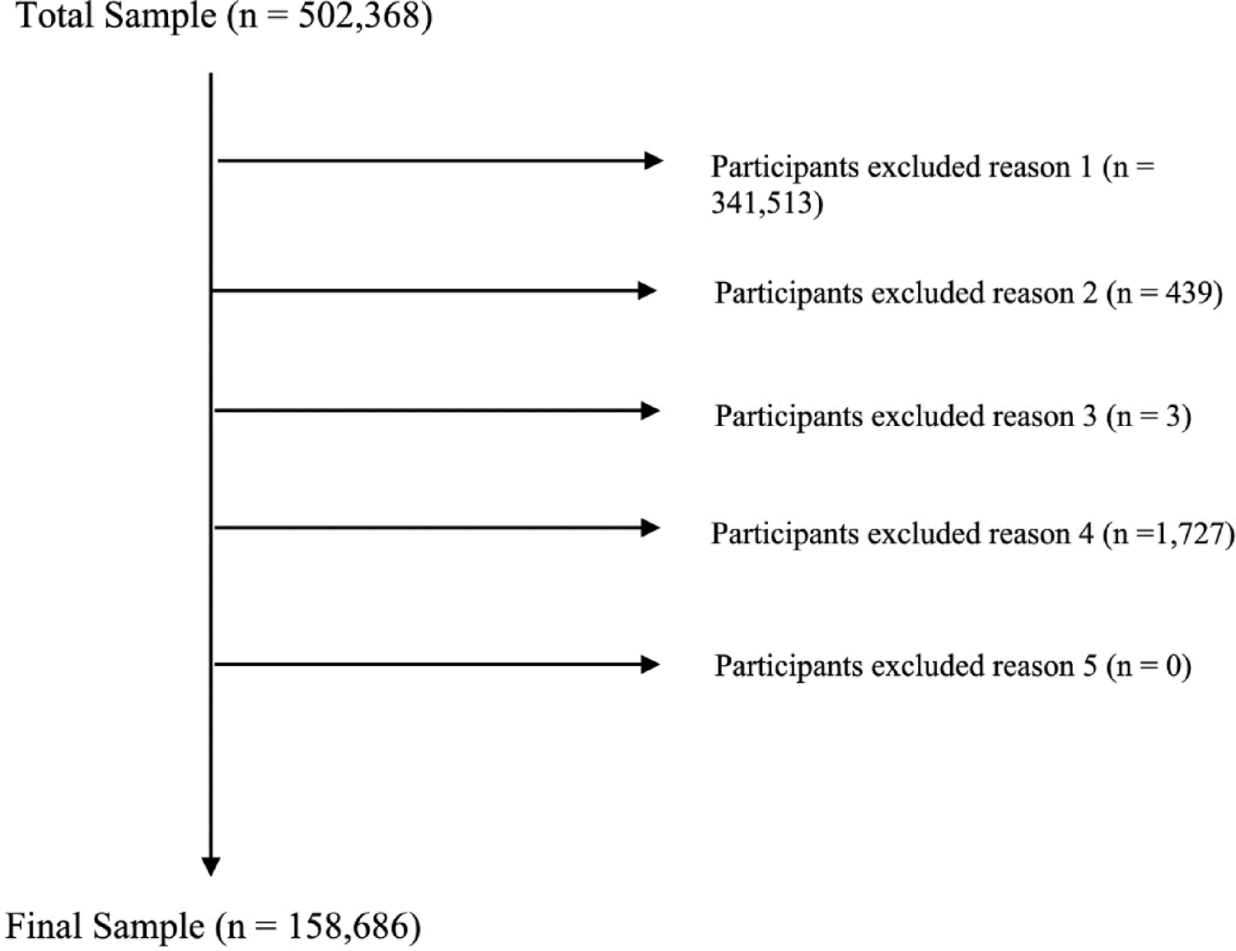
Reason for participant exclusion. Reasons for exclusion are as follows: 1. Having missing SRT data. 2. Have an SRT at ceiling (8 dB +). 3. They have a known diagnosis of PDD at the time of completing the assessment. 4. They go on to develop Alzheimer's or Parkinson's disease. 5. Missing covariate data

The final included sample was n = 158,686 (72,015 males; 86,671 females). See Fig. 1 for full breakdown of reasons for exclusion.

### Outcome measure

A probable diagnosis of PDD in accordance with The International Classification of Diseases-10 classification system (F02.3 Dementia in Parkinson's disease) was used as the primary outcome measure. This variable is obtained through participants’ linked hospital inpatient records. Within UK Biobank, this variable summarises all distinct ICD-10 diagnosis codes recorded for a participant across their linked hospital inpatient episodes, including codes documented in either the primary or secondary diagnostic position.

### Exposure measure

Hearing impairment was determined through the DTT speech-in-noise perception assessment. Briefly, 15 sets of three monosyllabic digits were presented against background noise via circumaural headphones. The background noise level varied adaptively after each triplet to estimate the signal-to-noise ratio at which 50% of the presented speech was recognised. The speech recognition threshold (SRT) was calculated as the mean signal-to-noise ratio for the last eight triplets. SRT is calculated independently for each ear. In accordance with prior studies, in this study hearing impairment was based on better ear performance.

Prior research indicates that within the UK Biobank sample, top-down central auditory processes have limited influence on DTT performance (Moore et al. 2014). However, additional research suggests that DTT performance is not only influenced by peripheral hearing sensitivity but also by central auditory function, and cognitive factors, including working memory, attention and processing speed (Dryden et al. 2017). Therefore, the SRT obtained from the DTT in the UK Biobank should be considered a mixed estimate, reflecting both peripheral and central hearing capabilities and cognitive function.

### Covariates

As this analysis is a pilot study, and we anticipated the incidence rate of PDD to be low, we elected to keep the statistical model as simple as possible, whilst acknowledging the fundamental limitations in doing so. Advancing age is a substantial independent risk factor for the incidence of Parkinson’s (Hoang et al. 2021), dementia (Hoang et al. 2021), and hearing impairment (Lin et al. 2011). Moreover, biological sex and level of educational attainment influence the occurrence of hearing impairment, with males and those with lower educational attainment being more likely than women and those with high educational attainment to have a hearing impairment (Nolan 2020; Lee et al. 2015). As such, the primary analysis included adjustment for age, biological sex, and educational attainment. Age was grouped into 5-year intervals, biological sex was coded as a categorical variable with two levels (male, female), and educational attainment, coded as a categorical variable with eight original response categories (O levels/ GCSE or equivalent; CSE or equivalent; A levels/AS levels or equivalent; NVQ or HND or HNC or equivalent; College or University degree; Other professional qualifications e.g.: nursing, teaching; None of the above; Prefer not to answer). All three covariates were included as stratification variables in the Cox proportional hazards model, allowing the baseline hazard to vary across age groups, sex, and education categories without estimating specific HRs or assuming proportional hazards for these variables.

### Data analysis

All statistical analyses were conducted using R version 4 (RStudio RT. 2020), and precisely adhered to the prespecified analysis plan detailed in the study protocol. The associated analysis scripts can be found in the OSF project file (Readman et al. 2024).

Descriptive statistics were used to describe the included sample. Whilst SRT data are not typically used to inform clinical decisions, to aid interpretability of the dataset, SRT data were also categorised and described in accordance with UK Biobank SRT norms (Dawes et al. 2014). Chi-squared tests of independence and independent samples t-tests were conducted to ascertain whether demographic characteristics significantly differ between people who go on to develop PDD and controls. To account for multiple comparisons, a Bonferroni correction was applied to all five between-group analyses, meaning the applied criterion was *p* < 0.01.

To evaluate the relationship between hearing impairment and PDD incidence a Cox Proportional Hazard Model adjusted for age was applied. Hypothesis testing for hearing loss was carried out using a two-sided alpha of 0.05. The *p* value, HR, and accompanying 95% confidence intervals (CIs) for the resulting model are reported.

### Public involvement

One person living with Parkinson’s and hearing impairment and one former unpaid carer of a person with Lewy body dementia (and CEO of a third sector dementia charity) were included as co-production public contributors in the research team. These individuals contributed to conceptualising the study, advising on interpretation of the findings, and dissemination. To ensure research integrity and avoid potential conflicts of interest, all data analysis and initial interpretation were conducted independently by lead author MRR prior to discussions with the public contributors. Their involvement ensured that the study and its implications of findings were grounded in the lived experiences of those affected by the Lewy body dementias and hearing impairment, while maintaining objectivity of the research process.

## Results

### Sample demographics

The mean age at the point of auditory test was 56.59 (*SD* = 8.15; Range 39–72). Overall, the sample contained slightly more females (54.62%) than males (45.38%), and people educated up to professional qualification level (See Table 1). In accordance with the UK Biobank SRT norms (Dryden et al. 2017), at baseline 110,868 (69.87%) people were classified as having ‘Normal’ hearing, 41,771 (26.32%) were classified as having ‘Insufficient’ hearing, and 6,047 (3.81%) were classified as having ‘Poor’ hearing. Although approximately one third of the total sample displayed some degree of hearing impairment, only 2.39% (*N* = 3,794) reported using a corrective hearing device (hearing aids or cochlear implants), with no difference in the proportion of usage being observed between those who went on to develop PDD (4.95%) and those who did not (2.39%, *p* = 0.17).

**Table 1:** Demographic characteristics of the analysed sample

	Full Sample (n = 158,686)	Participants with incident PDD (n = 101)	Controls (n = 158,585)	*p, χ2 *^*a*^
Age (years) [n (SD))	56.59(8.15)	63.92(4.54)	56.58 (8.15)	t (100.41) = -16.22, *p* < .001*
Biological Sex [n (%)]				χ^2^ (1) = 18.76, *p* < .001*
Female	86,671(54.62)	33 (32.67)	86,638(54.63)	
Male	72,015(45.38)	68 (67.33)	71,947(45.37)	
Educational Attainment [n (%)]				χ^2^ (7) = 13.59, *p* = .05
O levels/ GCSE or equivalent	20,846 (13.14)	11(10.89)	20,835 (13.14)	
CSE or equivalent	6,223 (3.92)	1 (0.99)	6,222 (3.93)	
A levels/AS levels or equivalent	11,376 (7.17)	9 (8.91)	11,367 (7.17)	
NVQ or HND or HNC or equivalent	18,135 (11.43)	12 (11.88)	18,123 (11.43)	
College or University degree	32,533 (20.50)	18 (17.82)	32,515 (20.50)	
Other professional qualifications e.g.: nursing, teaching	45,208 (28.49)	23 (22.77)	45,185 (28.49)	
None of the above	22,854 (14.40)	26 (25.74)	22,828 (14.39)	
Prefer not to answer	1,511 (0.95)	1 (.99)	1,510 (.95)	
Hearing Intervention [n (%)]				χ^2^ (1) = 1.85, *p* = .17
Yes	3,794 (2.39)	5 (4.95)	3,789 (2.39)	
No	154,892 (97.61)	96 (95.04)	154,796 (97.61)	
SRT [mean in dB (SD)]	-6.17 (1.45)	-5.59(1.65)	- 6.17(1.45)	t (100.1) = -3.51, p < .001*

^a^The p-values and χ2 documented here were obtained from independent sample t-tests and chi-squared tests of independence which examined whether demographic characteristics significantly differ between people with incident Parkinson’s and controls. *To account for multiple comparisons a Bonferroni correction was applied to the desired significance level (α) of 0.05. Given that 10 comparisons were conducted (across both this analysis and the analysis grouped by hearing impairment category), a required significance level of .005 was applied

Over a median follow up of 8.93 years (SD = 2.29 years), 101 cases of incident PDD were reported, equating to a crude incident rate of 6.4 cases per 10,000 people. People who went on to develop PDD were more likely to be older (*p* < 0.001) and male (*p* < 0.001) compared to controls. In a crude analysis, not controlling for age or sex, people who went on to develop PDD (*M* = -5.59, *SD* = 1.65) had significantly higher SRTs compared to controls (*M* =—6.17, *SD* = 1.45; t (100.1) = -3.51, *p* < 0.001), thus indicating that people who went on to develop PDD, on average, had poorer ability to detect speech in a background of noise. Of the 101 incident PDD cases, 50 (49.50%) people were classified as having ‘Normal’ hearing, 43 (42.57%) were classified as having ‘Insufficient’ hearing, and 8 (7.92%) were classified as having ‘Poor’ hearing. A greater proportion of people who went on to develop PDD were categorised as having some degree of hearing impairment, in particularly ‘Insufficient’ hearing, compared to those who did not go on to develop PDD (χ^2^ (2) = 20.62, *p* < 0.001).

### Survival analysis

Proportional hazards assumptions were evaluated using Schoenfeld residuals. No evidence of violation was found for the primary predictor SRT (χ^2^ = 0.66, df = 1, p = 0.42), and the global test indicated that the overall model satisfied the proportional hazards assumption (χ^2^ = 0.66, df = 1, p = 0.42).

**Fig. 2:**
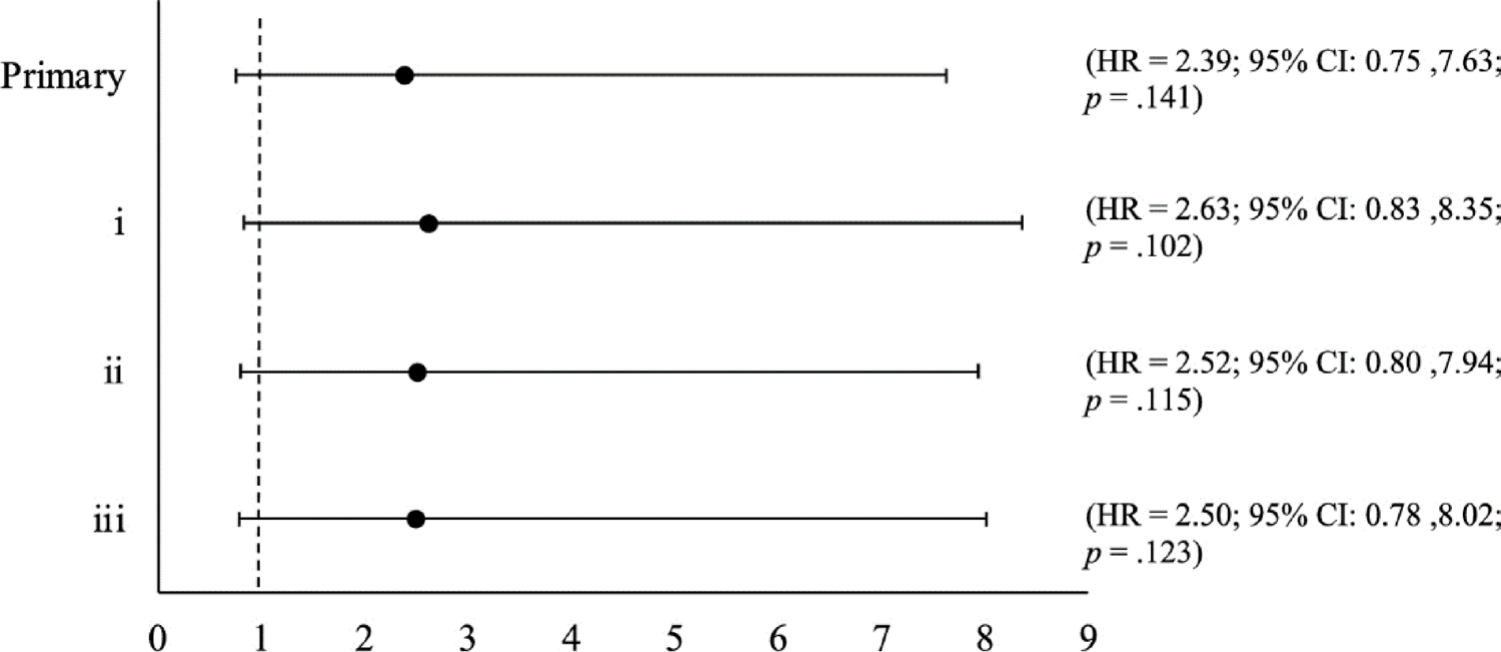
Forest Plot of the hazard ratio for the primary analysis and all additional sensitivity analyses. Primary is the stratified Cox Proportional Hazard model reported as the primary analysis and i-iii are the sensitivity analysis stratified Cox Proportional Hazard models. Full details of the models applied can be found below: [i] Predictor: Continuous SRT. Covariates: Age. [ii] Predictor: Continuous SRT. Covariates: Age, biological sex. [iii] Predictor: Continuous SRT. Covariates: Age ∗ educational attainment

For the given sample, after adjusting for age, biological sex and educational attainment, while the HR is compatible with a positive relationship between worsening SRT and PDD incidence (HR = 2.39), the degree of imprecision in estimates leaves it uncertain whether the excess risk of incident PDD was influenced by SRT (95% CI: 0.75,7.63; *p* = 0.141). Sensitivity analyses ((i) age only, (ii) age and biological sex, (iii) age and educational attainment) produced virtually unchanged results (see Fig. 2).

### Exploratory analysis

As an additional, not pre-registered, exploratory analysis, we analysed the influence of categorised hearing impairment on the risk of incident PDD. For the given sample, after adjusting for age, biological sex and educational attainment, “insufficient” (HR = 1.61, SE = 0.21; 95% CI: 1.07, 2.43, *p* = 0.024) but not “poor” (HR = 1.58, SE = 0.39; 95% CI: 0.74, 3.37, *p* = 0.236) hearing abilities significantly increased incident PDD risk. Sensitivity analyses produced virtually unchanged results (see supplementary materials Table S1 for full analyses).

## Discussion

This pilot study is the first to explore whether hearing impairment is a substantial risk factor for the incidence of PDD. We observed that while HRs were in the direction of a positive relationship between hearing impairment and PDD incidence, after adjustment for sex, age, and educational attainment, this relation was not statistically significant and should be considered exploratory. Importantly, this lack of statistical significance should not be interpreted as strong evidence against an association between hearing impairment and PDD, but may reflects the limited power and pilot nature of the present analysis.

Prior studies have estimated the incidence rate of PDD to be 2.5 cases per 10,000 (2.3 in men and 2.7 in women) (Savica et al. 2013). The estimated crude incidence rate in this sample, 6.4 cases per 10,000 people, was larger than prior estimates. However, an a priori power analysis indicated that a minimum of 200 events (cases of PDD) would be required to observe a significant relation between hearing impairment (with an estimated prevalence of 41.7% in the general population (NICE 2024) should one exist (Schoenfeld 1983). The present analysis included fewer events than this threshold, resulting in reduced precision and statistical power. Although this may have limited our ability to detect modest associations, we cannot exclude the possibility that the true effect is small or null in this sample. Accordingly, these findings should be interpreted as exploratory and hypothesis-generating and do not negate the broader body of evidence linking hearing impairment with neurodegenerative outcomes (Schrag et al. 2023, Lai et al. 2014, Simonet et al. 2022, Readman et al. 2025a, Readman et al. 2023, Readman et al. 2025b, Livingston et al. 2024).

While acknowledging the fact that these conclusions should be treated as preliminary and exploratory, these observations contribute to the body of literature considering hearing impairment as a risk factor for dementia. While substantial imprecision in estimates leaves it uncertain whether the excess risk of incident PDD was influenced by SRT, the observed HR directionally aligns with previously observed positive relationships between hearing impairment and all-cause dementia incidence (Readman et al. 2025b; Livingston et al. 2024), though this should be interpreted cautiously. Furthermore, while imprecise, the findings of our primary analysis mirror the previously observed positive relationship between hearing impairment, both derived through SRT (Readman et al. 2025a) and clinical diagnosis (Lai et al. 2014; Simonet et al. 2022), and Parkinson’s incidence. It is, however, important to note that not all prior literature is consistent. Specifically, some studies, that focus on specific dementia subtypes, have observed hearing impairment (yes/no) is not associated with increased risk of incident vascular dementia, for example (Readman et al. 2025b; Yu et al. 2024). Therefore, while the HRs obtained in this study are consistent with a positive relationship between hearing impairment and dementia incidence, they do not establish a definitive effect, and the literature as a whole may indicate that the relationship between hearing impairment and dementia is perhaps not as straight forward as hearing impairment increases the risk of incident dementia, regardless of dementia subtype. Rather, it may be that the relationship between hearing impairment and dementia is more nuanced and the magnitude of the relationship may depend upon factors including the specific dementia subtype. Thus, future research should continue to consider the relationship between hearing impairment and specific dementia subtypes.

It is, however, important to note that while the HR from the primary analyses directionally align with previously observed positive associations between hearing loss and all-cause dementia incidence (Readman et al. 2025b; Livingston et al. 2024), the exploratory categorical analyses are inconsistent with earlier findings. Specifically, prior literature has consistently demonstrated a dose–response relationship, where increasing severity of hearing impairment is associated with a higher risk of dementia (Kumar and Singh 2015). However, here we observed that when hearing impairment was categorised in accordance with UK Biobank SRT norms (Dawes et al. 2014), only the ‘Insufficient’ hearing category, and not the ‘Poor’ category, was significantly associated with PDD risk compared to ‘Normal’ hearing. Notably, for the dataset analysed 50 (49.50%) people were classified as having ‘Normal’ hearing, 43 (42.57%) were classified as having ‘Insufficient’ hearing, and 8 (7.92%) were classified as having ‘Poor’ hearing. Therefore, the absence of statistical significance for the ‘poor’ category likely reflects limited precision rather than evidence against increasing risk with greater impairment. Accordingly, these categorical findings should be considered descriptive and hypothesis-generating rather than indicative of threshold or non-linear effects. Future studies with larger samples are required to robustly evaluate potential dose–response relationships between hearing impairment and PDD.

These findings may offer preliminary insights into potential mechanisms linking hearing loss and dementia. However, given the pilot nature of this study and the uncertainty around effect estimates, any mechanistic interpretations should be regarded as speculative and contextual rather than confirmatory. Dementia subtypes share several commonalities; they all feature cognitive symptoms affecting memory, thinking, and social abilities. Furthermore, dementia subtypes oftentimes have co-existing pathology. For example, AD pathology, including neurofibrillary tangles and extracellular amyloidal protein deposits (Kumar and Singh 2015), is commonly observed in Lewy body dementias (Tan et al. 2025) including PDD (Prajjwal et al. 2024). Despite these commonalities, differences are seen at the level of symptom presentation and neuropathology. For example, PDD is neuropathologically characterised by aggregates of α-Synuclein Lewy bodies in limbic and neocortical areas of the brain which do not typically occur in AD pathology (Kouli et al. 2020). In relation to symptoms, PDD features motor, behavioural and psychiatric symptoms, which are not typically seen in other dementia subtypes (Degirmenci et al. 2023; Goetz et al. 2008). If it is the case that hearing loss is a significant risk factor for the incidence of multiple dementia subtypes, this may suggest that the underlying mechanism linking the two is common across different forms of dementia. This could be a marker of generalised neurodegeneration, for example mitochondrial oxidative stress (Mao 2013; Bennett et al. 2009), which would thereby support the common cause hypothesis (Griffiths et al. 2020). The positive relationship between hearing impairment and the incidence of alternative neurogenerative diseases, such as Parkinson’s (Lai et al. 2014; Simonet et al. 2022; Readman et al. 2025a), further supports this assumption. However, as the present study did not analyse the relation between hearing loss and dementia across multiple subtypes, nor control for the potential influence of markers of generalised neurodegeneration and comorbidities, this interpretation is speculative. Large-scale studies that examine hearing loss as a risk factor across distinct dementia subtypes within the same cohort, using consistent covariate adjustments, are needed to clarify these relationships.

Within this study the presence of hearing impairment was based on speech-in-noise perception, estimated through the DTT. While prior evidence indicates that top-down, central auditory and cognitive processes have limited effects on DTT performance in the UK biobank sample (Moore et al. 2014; Dryden et al. 2017), it would be an oversimplification to assume that performance on the DTT relies only on peripheral auditory acuity. Indeed, in a meta-analysis of 253 studies, Dryden et al. (2017) observed that inhibitory control, working memory, episodic memory, crystalised IQ, and processing speed all significantly correlate with speech-in-noise perception capabilities. As such, poor performance on the DTT may reflect not only hearing deficits but also cognitive changes. Specifically, poor performance on the DTT could itself be an early manifestation of the cognitive decline inherent in prodromal PDD, rather than purely reflecting peripheral hearing loss. Therefore, the observed association might reflect subclinical cognitive or central auditory processing deficits characteristic of evolving PDD, rather than an association between peripheral hearing loss and PDD. This supports the plausibility of which would support the reverse causation, in which prodromal PDD impairs auditory processing (Griffiths et al. 2020). In contrast, pure tone audiometry (PTA) assessment is thought to depend primarily upon the health of the hair cells (Pickles 1988), thereby providing a more direct measure of peripheral auditory sensitivity with less impact of central auditory processes. While PTA data are not available in the UK Biobank, future studies should consider incorporating both PTA and speech-in-noise assessments to explore the relationship between PTA and PDD incidence. This would help disentangle peripheral versus central auditory contributions and further clarify the potential for reverse causality.

If these findings hold over larger and alternative cohort studies, they may pose several important practical implications. Specifically, the finding that hearing impairment may be a risk factor for the incidence of dementia, regardless of the specific subtype, may reinforce the need for the consideration of hearing impairment in the clinical management of all types of dementia. Moreover, our findings may have implications of public health messaging. Specifically, they may indicate that it is appropriate that population-level dementia risk reduction messaging focused on hearing impairment takes a blanket approach (i.e. referring to all-cause dementia), rather than being tailored to specific dementia subtypes. However, these findings require further substantiation before recommendations of alterations to public health messaging are made.

This study is not without its limitations, one being the low PDD incidence rate in the final analytical sample (n = 101). This study was a prospective cohort study employing the UK Biobank dataset, and as such we are bound by the limitations of the dataset cohort. Therefore, further studies, employing alternative datasets, with higher PDD incidence rates are required to substantiate the non-significant findings of a relation between hearing impairment and PDD obtained here. It is, however, important to highlight that most biomedical datasets employed in analyses of dementia and its associated risk factors do not collect dementia subtype data (Readman et al. n.d.). Therefore, relying on secondary data to explore risk factors associated with specific, rarer, dementia subtypes may be somewhat problematic.

A second limitation relates to the ascertainment of PDD diagnosis. Within this study we ascertained PDD diagnosis by the presence of an ICD code pertaining to dementia in Parkinson’s in linked hospital inpatient episodes, including codes documented in either the primary or secondary diagnostic position. Prior evidence demonstrates substantial discordance between patient and carer perceived and objectively measured cognitive difficulties in Parkinson’s (Copeland et al. 2016). Given that receipt of a clinical diagnosis of PDD first requires patient/carer identification and complaint to a health professional, it may be that the true prevalence of PDD in the UK Biobank dataset is underestimated. This may have been further compounded by the decision to exclude participants that went on to develop Parkisnon’s without dementia from the analysis. Therefore, future studies should consider using standardised cognitive assessment (e.g. the Addenbrookes cognitive examination) to ascertain the presence of PDD rather than relying upon the presence of an ICD-9/10 diagnosis in linked health records.

Furthermore, the final analytical sample contained more females than males and people educated up to a professional qualification level than those with no or lower qualifications. Individual characteristics, including education and biological sex, are known to influence dementia risk but also access to a dementia diagnosis (Giebel 2024). For example, women and people with lower educational attainment are more likely to go on to develop dementia than males and people with higher educational attainment (Livingston et al. 2024). Moreover, people with higher educational level may be more likely to receive comprehensive dementia diagnosis workups and have a higher likelihood of obtaining a specific dementia subtype diagnosis than people with lower educational levels (Hoang et al. 2021). Given that the final analytical sample included more females than males and people educated up to professional qualification level, caution should be applied when generalising these findings to wider populations.

Whilst age, biological sex, and educational attainment were controlled for as covariates in this study, additional established risk factors for both PDD and hearing impairment incidence, such as cardiovascular disease, diabetes, depression, smoking, and physical inactivity, were not controlled for. This decision was in driven by the relatively low number of incident PDD cases and the missingness of such covariate data, which constrained the number of covariates that could be included without compromising statistical stability. As such, residual confounding cannot be ruled out, and uncontrolled clinical and lifestyle factors may either inflate or attenuate the observed association between DTT and PDD incidence. Future studies aiming to replicate these findings in alternative samples would benefit from incorporating a broader set of clinical and lifestyle risk factors as covariates to strengthen causal inference and reproducibility.

A final limitation, relates to the ascertainment of hearing impairment through the DTT. The DTT reflects peripheral and central auditory function, and cognitive processing (Moore et al. 2014; Dryden et al. 2017). As such reverse causality cannot be ruled out and the observed association between DTT performance and PDD incidence should be interpreted as correlational rather than causal. Furthermore, although the DTT is widely used in large-scale epidemiological studies, it is not a standardised audiometric assessment routinely employed in clinical practice. In contrast PTA is the conventional clinical benchmark for assessing peripheral auditory function and characterising hearing profiles. The absence of PTA data therefore precludes the validation of DTT derived measures against standardised clinical hearing loss profiles. Consequently, the clinical applicability and comparability of these findings with other studies using conventional audiometric assessments is limited. Future studies should incorporate PTA alongside DTT measures to improve reliability and interpretability of the findings.

## Conclusion

To conclude, the present pilot study observed HRs suggestive of a positive relationship between baseline hearing impairment and PDD incidence; however, imprecision in estimates and lack of significance leaves it uncertain whether the excess risk of incident PDD is influenced by continuous SRT. Furthermore, when SRT is categorised in accordance with UK Biobank norms (Lee et al. 2015), only the ‘insufficient’ hearing category but not ‘poor’ hearing category was associated with increased risk of incident PDD. Given the limited sample size and statistical power, these findings should be considered descriptive and hypothesis-generating and further studies using alternative datasets with larger numbers of incidence PDD cases are required to substantiate these findings before firm conclusions are drawn. If further supported, these findings have significant implications for clinical practice and public health messaging.

## Supplementary Information

Below is the link to the electronic supplementary material.

Supplementary file1 (DOCX 20 KB)

## Data Availability

Data supporting this study are available from the UK Biobank at ukbiobank.ac.uk. Access to the data is subject to approval.
