# Supplementary material for: Parkinson’s disease dementia and hearing impairment, are they related? A UK biobank pilot analysis
Source: Exp Brain Res. 2026 Apr 9;244(5):85. doi: 10.1007/s00221-026-07282-1 (PMC13065560; doi:10.1007/s00221-026-07282-1)
Supplement: Supplementary file 1 — Supplementary file1 (DOCX 20 KB) [file 221_2026_7282_MOESM1_ESM.docx]

**Supplementary Materials**

**Contents**

1. Sensitivity analyses for exploratory analysis 2
2. Sensitivity analyses for exploratory analysis

**Table S1.**

Sensitivity analyses for exploratory analysis.

| Covariates | Exposure | HR (Standard Error) | Upper and Lower 95% CI | *p* |
| --- | --- | --- | --- | --- |
| Age, biological sex, educational attainment | Insufficient hearing  Poor hearing | 1.61 (.21)  1.58 (.39) | 1.07, 2.43  .74, 3.37 | .024  .236 |
| Age | Insufficient hearing  Poor hearing | 1.61 (.21)  1.66 (.38) | 1.07, 2.43  .78, 3.53 | .022  .186 |
| Age, biological sex | Insufficient hearing  Poor hearing | 1.62 (.21)  1.61 (.38) | 1.07, 2.44  .76, 3.43 | .022  .186 |
| Age, educational attainment | Insufficient hearing  Poor hearing | 1.60 (.21)  1.63 (.38) | 1.06, 2.42  .76, 3.43 | .025  .207 |
